# Introducing the contextual digital divide: Insights from microscopic anatomy on usage behavior and effectiveness of digital versus face‐to‐face learning

**DOI:** 10.1002/ase.70010

**Published:** 2025-02-28

**Authors:** Morris Gellisch, Juliane Cramer, Justin Trenkel, Franziska Bäker, Martin Bablok, Gabriela Morosan‐Puopolo, Thorsten Schäfer, Beate Brand‐Saberi

**Affiliations:** ^1^ Center for Medical Education Ruhr‐University Bochum Bochum Germany; ^2^ Department of Anatomy and Molecular Embryology, Institute of Anatomy, Medical Faculty Ruhr University Bochum Bochum Germany

**Keywords:** digital anatomy, e‐learning, histology, learning, teaching of anatomy

## Abstract

In recent years, there has been a growing trend toward the digitization of education, highlighting the need to understand how different learning modalities impact medical student outcomes. This study analyzes user behavior among medical students in Microscopic Anatomy, focusing on preferences for digital versus face‐to‐face guided self‐study. A cohort of 684 students participated in Microscopic Anatomy courses, choosing between digital and face‐to‐face guided self‐study sessions. Student participation was systematically tracked, capturing detailed data on the frequency and duration of engagement with each self‐study modality. Quantitative measures of academic performance were collected, and user behavior was analyzed to identify trends in learning outcomes. The majority preferred face‐to‐face sessions, with attendance increasing over the semester. By the end, 78.44% found hands‐on engagement with microscopes more effective and engaging than digital alternatives. Students attending in‐person sessions achieved superior academic outcomes, with a failure rate of 13.69% compared to 22.04% among nonattendees. Preferences were influenced by direct tutor interactions (61.38%) and peer engagement during free microscopy sessions (74.25%). Additionally, 46.71% reported higher motivation when studying in the histology lecture hall compared to at home. These findings reveal a ‘Contextual Digital Divide’, where digital learning may not fully meet educational needs in contexts requiring hands‐on experiences and direct interaction. Our results highlight the need to reinforce face‐to‐face teaching in specific contexts to ensure an effective learning experience. Future research should build on these insights to better understand situations where digital methods may fall short of learners' needs.

## INTRODUCTION

Histology, also known as Microscopic Anatomy, holds profound significance in medical education for its key role in understanding the complex structure and function of tissues and organs at a cellular level. By examining tissues under the microscope, medical students gain invaluable insights into the physiological processes that underpin health and disease, laying the groundwork for comprehending pathology during further clinical studies.^1^, ^2^ In addition to its foundational importance in understanding tissue and organ structures, histology education increasingly intersects with advancements in computational technologies. Deep learning, for instance, has demonstrated significant potential in histopathology by enabling precise analysis of complex tissue patterns, such as predicting tumor molecular genetics and automating diagnostic workflows.^3^, ^4^ These advancements underscore the modern relevance and adaptability of histology, highlighting that a fundamental understanding of its principles is essential to effectively leverage emerging technologies in diagnostic and research contexts.

In recent years, the landscape of medical education has undergone a dynamic transformation with the increasing adoption of digital technologies.^5^, ^6^ The convergence of innovative teaching tools and the ubiquitous accessibility of online resources has revolutionized how students engage with complex anatomical concepts. Against the backdrop of this digital revolution, the COVID‐19 pandemic further accelerated the shift toward virtual learning environments, prompting institutions worldwide to explore remote teaching modalities.^7^, ^8^, ^9^ The global health crisis necessitated rapid adaptations in educational delivery to comply with social distancing measures and ensure the continuity of learning amidst widespread disruptions. Consequently, institutions worldwide swiftly embraced digital innovations in medical education, possibly further incorporating them in the “post‐COVID era”.^8^

The trends of digitalizing medical education methods can particularly be observed in Microscopic Anatomy.^10^ Tauber et al. found that students benefited from e‐learning strategies in histology.^11^ They reported an enhanced microscopy experience due to higher comfort of viewing virtual slides, an increase in detail perception, and fewer technical problems.^11^ Moreover, students—previously engaged in digital histology learning environments—performed better in subsequent histological testing.^11^ On the contrary, virtual microscopy has been criticized as a loss of opportunity for students to learn the utilization of traditional microscopes, possibly affecting students’ practical skills and critical thinking abilities alongside curiosity and motivation.^10^, ^12^ An integrative review by Joaquim et al. therefore suggests the interactive combination of traditional educational methods and digital technologies in order to impact academic performance and histological skills as effectively as possible.^13^

At the Institute of Anatomy at Ruhr University Bochum, the course of Microscopic Anatomy spans three semesters and is divided into three sub‐sections. Microscopic Anatomy I focuses on cellular biology and fundamental tissue types. Microscopic Anatomy II covers the histology of internal organs and incorporates pathological specimens to bridge foundational knowledge with clinical applications. Microscopic Anatomy III examines the histology of the peripheral and central nervous system and sensory organs. Practical sessions, guided by lecturers, allow students to examine tissue sections under the microscope and are complemented by lectures throughout the semesters. Apart from the curriculum‐oriented lectures and courses, the Institute of Anatomy at Ruhr University Bochum offered two additional voluntary study options for medical students learning histology—the digital guided self‐study offer and the face‐to‐face guided self‐study offer—aiming to deepen their comprehension of Microscopic Anatomy and to resolve open questions. The digital guided self‐study offer included the online virtual microscopy tool called MyMi.mobile (Ulm University, Ulm, Germany, accessed on June 1, 2024), which includes annotations and further explanations of microscopic structures. It contains the same histological specimens that are available during the practical courses and, in addition, includes specimens from other university centers. Apart from the possibility of virtually inspecting the histological specimens with annotations, the students were also offered an additional online forum, moderated by near‐peer histology tutors. This digital guided self‐study offer provided various tools, including an open forum for discussing histological questions with study peers as well as an anonymous questionnaire tool in which trained histology tutors answered questions individually. Students were also able to upload pictures or drawings from histological specimens into the described online forum, laying the groundwork for further discussions. To illustrate the educational value of drawing in histology learning,^14^ we have included a figure in the supplementary materials showcasing four examples of student‐created drawings (Appendix A). These drawings demonstrate how students engage with and internalize histological structures through active observation and replication.

Near‐peer tutorship or mentoring in general can be defined as a “formal relationship in which more qualified students guide immediate junior students”.^15^ This popular method aims to boost the engagement of students and has the potential to be as effective as faculty teaching.^15^, ^16^ Near‐peer tutors are described to be more aware of students’ level of understanding and provide a comparably low threat learning environment, therefore they were appointed to manage the online forum as well as to support face‐to‐face study options.^15^ Further, given that interactive learning elements have been shown to enhance student engagement but also tend to elevate anxiety levels,^17^ we incorporated near‐peer tutor systems into the digital learning environment in place of lecturers to mitigate this effect.

As the face‐to‐face guided self‐study option, weekly time slots for individual in‐person microscopy sessions were offered by the Institute of Anatomy. These sessions took place in the histology lecture hall, where students were provided with a microscope and all histological specimens of the course. During these sessions, near‐peer histology tutors were available for questions and further discussions in a face‐to‐face setting. The students were also encouraged to work on voluntary histological drawings or sketches during these sessions. Studies have shown that drawing histological specimens improved the engagement of the students with the practical material.^18^ The students observed the photomicrographs more thoroughly, showed enhanced knowledge retention, and achieved higher scores in practical assessments.^18^, ^19^, ^20^

Thus, the students were able to choose between two learning environments: studying histological specimens at home using virtual microscopy tools with feedback via an online forum or participating in in‐person sessions in the histology lecture hall, receiving feedback face to face from near‐peer histology tutors.

To conclude, two near‐peer learning options for Microscopic Anatomy were offered by the Institute of Anatomy, comprising a digital format and a face‐to‐face format. The central objective of this study is to explore the respective patterns of usage and the comparative effectiveness of both the digital guided self‐learning offer and the face‐to‐face self‐learning offer. This study builds on the evolving concept of the digital divide, traditionally described across three levels: disparities in access to IT (‘digital access divide,’ first level), the ability to use it effectively (‘digital capability divide,’ second level), and the outcomes achieved through its use (‘digital outcome divide,’ third level).^21^ By adapting this framework, we introduce the ‘Contextual Digital Divide,’ a concept that highlights the gap between students’ educational needs and the capacity of digital tools to meet those needs in specific learning contexts.

## MATERIAL AND METHODS

### Study design

**FIGURE 1:**
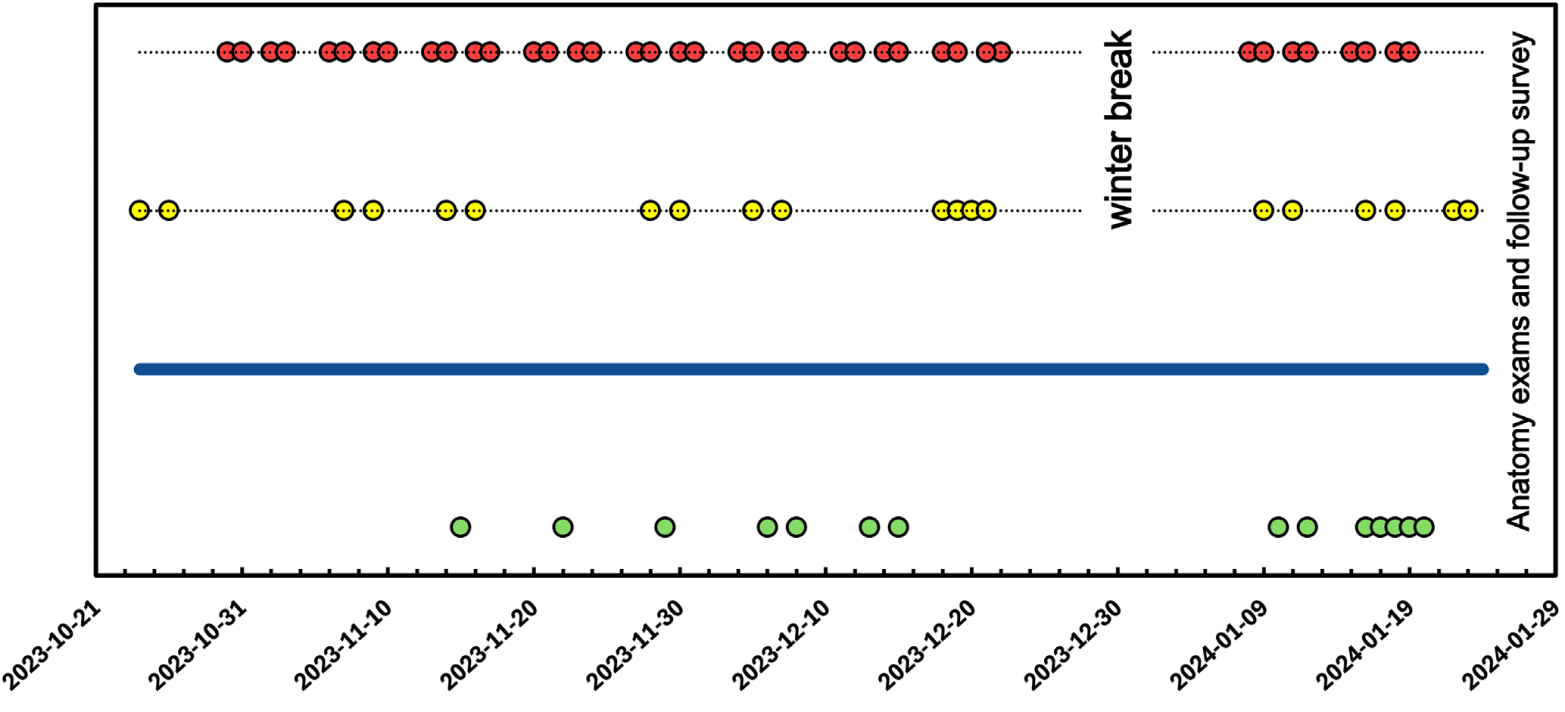
This flow chart presents the sequence of anatomy learning activities from October 21, 2023, to January 29, 2024. Microscopic Anatomy I sessions (red circles) and Microscopic Anatomy III sessions (yellow circles) occurred regularly, alongside face‐to‐face guided self‐study sessions (green circles). A continuous digital guided self‐study offer (blue line) was available throughout the semester. The timeline includes a winter break, followed by anatomy exams and a follow‐up survey to gather student feedback on learning experiences.

This study was conducted in accordance with the ethical standards outlined in the Declaration of Helsinki and approved by the Ethics Committee of the Professional School of Education at Ruhr University Bochum (Approval Number: EPSE‐2023‐007, issued on 21st September 2023). The practical course in Microscopic Anatomy at Ruhr University Bochum, Germany, served as the setting for this study. First‐ and third‐semester medical students participated in a series of regular in‐person sessions, specifically within the ‘Microscopic Anatomy I’ or ‘Microscopic Anatomy III’ course, held in the university's histology lecture hall. In addition to these face‐to‐face classes, students were given the freedom to choose between two guided self‐study options—either a digital format or an in‐person format (Figure 1). Data on attendance and engagement with these learning modalities were systematically collected throughout the winter term of 2023/2024. At the end of the semester, all students completed a standardized follow‐up questionnaire to provide feedback on their experiences with the different learning environments. The primary aim of this study was to analyze usage patterns and evaluate the effectiveness of the guided self‐study offerings in both settings. By comparing students’ chosen formats and correlating these preferences with their final exam scores, this study seeks to uncover distinctions between digital and in‐person learning experiences when students are free to select their preferred modality. To provide additional transparency regarding the assessment structure in Microscopic Anatomy, we have included a table in the supplementary materials with examples of typical exam questions from the final knowledge test (Appendix B). These questions are designed to assess a range of competencies, from basic identification and understanding of tissue types to more advanced aspects of structural and functional knowledge. For instance, students may be asked to identify specific features of smooth muscle cells, describe the components of the juxtaglomerular apparatus, or recognize key characteristics of tissue organization.

### Participants

Including all medical students from the first and third semesters, we aimed at gathering comprehensive information about their experiences, usage behavior, and attendance in optional self‐study offerings, particularly at the beginning of their medical education. The inclusion criteria required participants to be properly enrolled first‐ or third‐semester students in human medicine at Ruhr University Bochum.

A total of 684 participants took part in this study: participants in this study included first‐semester medical students (345 in total; 110 males: mean age = 20.36 ± 2.81 years; 235 females: mean age = 20.29 ± 2.39 years) and third‐semester medical students (339 in total; 116 males: mean age = 22.16 ± 3.53 years; 223 females: mean age = 21.39 ± 3.01 years).

The students attended the respective courses of Microscopic Anatomy I (first‐semester students) or II (third‐semester students) and had the option to utilize additional educational resources on a voluntary basis.

### The digital guided self‐study offers

The digital guided self‐study offer centers on an interactive online forum where students can ask questions, upload digitized specimen screenshots, and receive feedback from trained near‐peer tutors proficient in histology. This forum allows students to engage in subject‐specific discussions, participate in collaborative quizzes, and anonymously ask questions that tutors or peers can answer in either private or public conversations. Students can also upload histological drawings for feedback and evaluation, enhancing their learning comprehension.

One of the key tools available for digital learning is “MyMi.mobile” provided by the Institute of Molecular and Cellular Anatomy, Ulm University (https://mymi.uni‐ulm.de, accessed on June 1, 2024). This tool enables students to navigate through all histological specimens available at the Institute of Anatomy at Ruhr University Bochum, as well as additional specimens from participating universities, including ETH Zurich, University of Zurich, MH Hannover, University of Freiburg, University of Münster, and University of Ulm. To provide digital microscopy for the students, all histological specimens were scanned using a Zeiss Axio Scan.Z1 Slide Scanner and processed with ZEN Blue software, version 2.3 (Carl Zeiss AG, Oberkochen, Germany). MyMi.mobile offers an intuitively easy‐to‐use interface, allowing navigation of the images via mouse movements, touchscreen, or a slider control. Students can access this virtual microscopy tool using various digital devices, including tablets and laptops.

### The face‐to‐face guided self‐study offer

The foundation of the face‐to‐face guided self‐study offer was the access students had to the histology lecture hall. In this setting, students could find every single specimen on glass slides, alongside microscopes equipped for both analog use and digital display. Here, each student was supplied with a Leica DM500 microscope (Leica Microsystems GmbH, Wetzlar, Germany) equipped with a Leica ICC50 W camera module (Leica Microsystems GmbH, Wetzlar, Germany), facilitating the observation of histological specimens on both a monitor and through the eyepiece, with the aid of Leica LAS EZ microscope software for on‐screen microscopy. The ability to display specimens on the screen facilitates group discussions, as everyone can view the same area simultaneously and engage in collaborative learning. Students had the opportunity to visit the histology lecture hall weekly to use these microscopes independently. The digital display option also enabled them to take screenshots and discuss specific areas of the slides with their peers. Additionally, near‐peer tutors were available throughout the entire session to provide support, answer questions, and foster discussions. This ensured that while students were self‐studying, they always had the opportunity to seek guidance and engage in meaningful conversations with their study group and tutors.

### Assessment of user behavior and follow‐up questionnaire

User behavior was assessed by measuring the frequency of interactions within the digital guided self‐study offer and the frequency and duration of use of the face‐to‐face learning offer.

Upon completion of the semester, students were asked to evaluate their usage behavior through a retrospective questionnaire. The items for this questionnaire were developed by an expert panel, including one anatomy professor, three anatomy lecturers, and five student representatives. This collaborative approach allowed us to design a questionnaire uniquely suited to our specific educational context, capturing insights that might be overlooked by standardized tools. While tailored to our needs, this customization means the questionnaire has not undergone formal validation in other settings, reflecting its strength as a context‐specific instrument. The follow‐up questionnaire was administered anonymously, and participation was entirely voluntary. Responses were collected online using the university's secure Moodle platform to ensure data privacy. The questionnaire included structured items to assess different aspects of the learning offers. The first set of items, Q1: “Digital guided self‐study forum (on Moodle),” comprised five questions aimed at evaluating difficulties with the online forum, focusing on efficiency, handling, and communication: (1) I was uncertain about the effectiveness of digital communication in addressing my queries regarding microscopic anatomy; (2) I preferred posing questions in person as I anticipated clearer and more direct responses thereby; (3) The forum provided ample opportunity for in‐depth discussions on microscopic anatomy; (4) Technical barriers or lack of familiarity with the digital forum deterred me from utilizing it; and (5) I had the impression that my questions were too specific or complex to effectively discuss them in a digital format.

The second set, Q2: “Digital Microscopy,” included five questions related to the utilization patterns and experiences with the online digital microscope: (1) the digital representation of specimens was of high quality and detailed; (2) the software for digital microscopy was intuitive and easy to use; (3) I effectively utilized digital specimens to accommodate my own learning pace and needs; (4) the ability to annotate and save digital specimens myself enhanced my learning experience; and (5) technical issues or limitations hindered my learning experience with digital microscopy.

The third set, Q3: “Face‐to‐face guided self‐study offer,” consisted of five items that gauged students’ opinions on the equipment in the lecture hall and its effectiveness in their learning process: (1) the direct interaction with tutors or lecturers in person significantly contributed to my understanding of microscopic anatomy; (2) learning at the microscope in the on‐site histology lecture hall provided me with a more practical and engaging learning experience; (3) engaging in free microscopy sessions in person facilitated exchange and discussion with my peers; (4) I found the physical presence in the histology lecture hall more motivating than learning from home; and (5) the quality and availability of specimens in the on‐site histology lecture hall were superior to the digital alternatives.

The fourth set, Q4: “Histological Sketches,” focused on students who created sketches, evaluating their perceptions of this activity as a supplemental learning tool: (1) I created my own drawings of histological specimens to deepen my understanding of microscopic anatomy; (2) creating drawings helped me better recognize and understand important details and structures of the histological specimens; and (3) generating my own drawings was an effective method for enhancing my long‐term memory of microscopic anatomy. Each item was ordinally scaled with response options: “disagree,” “partly agree,” and “fully agree.”

In addition to these structured items, the questionnaire provided a text field where students could express their ideas and criticisms regarding the utilization of the additional learning resources. This allowed for the qualitative assessment of general statements and provided deeper insights into students’ experiences and perceptions.

## RESULTS

This section presents the findings on user behavior and the evaluation of first‐ and third‐semester medical students’ usage experiences regarding the face‐to‐face and digital guided self‐study offers in Microscopic Anatomy. The analysis includes descriptive statistics on the frequency and duration of use, which were measured throughout the semester. Additionally, the results were analyzed to identify usage patterns and their correlation with performance in the final examinations of Microscopic Anatomy I and II. Finally, the item sets from topics Q1–Q4 were analyzed to gain insight into why certain learning environments were preferred, thereby providing context to the quantitative data on usage behavior.

**FIGURE 2:**
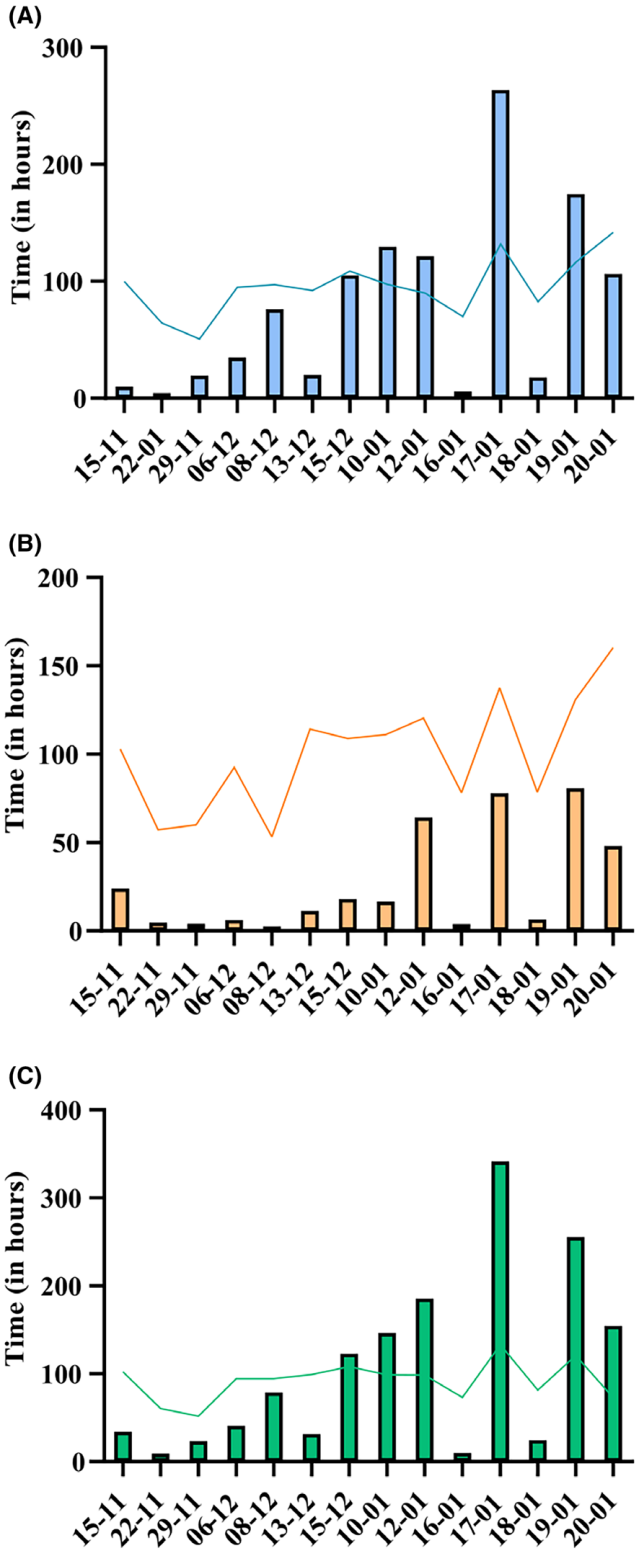
Total and mean time spent at the lecture hall. The bars represent the total time in hours. The line indicates the mean time spent in minutes. (A) First‐semester students. (B) Third‐semester students. (C) Combined data for students from both semesters.

For the face‐to‐face guided self‐study offer, from November 2023 to January 2024, fourteen regular sessions were scheduled, providing students the opportunity to spend time in the histology lecture hall under the supervision of trained tutors. In reference to this guided self‐study offering, attendance data indicates a rising trend in participant numbers as the semester progresses, particularly as the final exam approaches. During the first available session on November 15, 2023, a total of 20 students attended, comprising 6 first‐semester students and 14 third‐semester students. By the session held in the week prior to the final examination on January 17, 2024, attendance had risen to 154 students, with 120 first‐semester students and 34 third‐semester students. On average, 57 students attended each session, with 43.36 (76.74%) enrolled in Microscopic Anatomy I and 13.14 (23.26%) enrolled in Microscopic Anatomy II (Figure 2).

Considering both individual and average time spent in the histology lecture hall, a significant increase was observed. For first‐semester students, the average duration of stay at the initial session was 1.67 h, which increased to 2.36 h by the final session. Similarly, third‐semester students initially spent an average of 1.72 h per session, increasing to 2.67 h by the end of the semester. Overall, the average time spent across all sessions was 1.53 h for first‐semester students and 1.60 h for third‐semester students (Figure 2).

**FIGURE 3:**
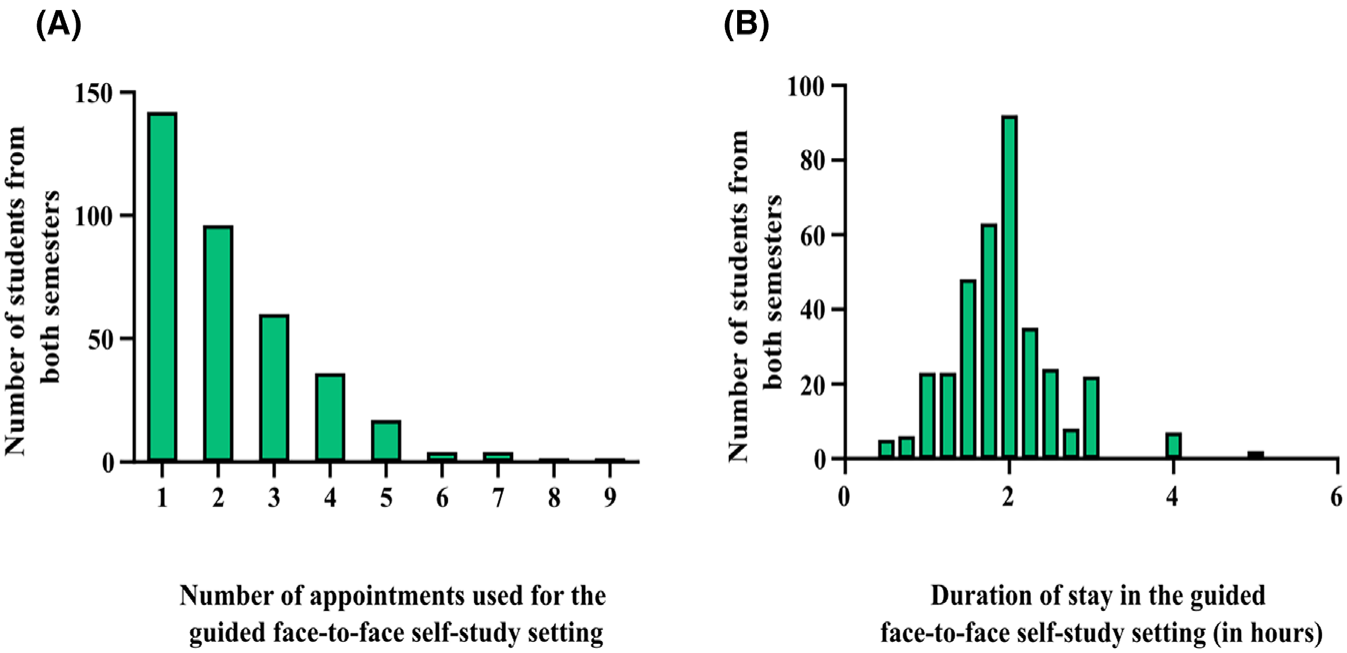
(A) Bar chart showing the distribution of the number of appointments attended by students, with a majority attending one or two appointments and a sharp decline in attendance for higher numbers of appointments. (B) Bar chart displaying the distribution of the duration of stay per session, with an almost normal distribution centered around 2 h and outliers extending to 4 and 5 h.

The majority of students utilized the histology lecture hall for one or two appointments, with the number of attendees showing an almost exponential decline as the number of appointments increased. This trend culminated in the lowest number of students attending nine appointments (Figure 3A). Regarding the duration of stay in the face‐to‐face guided self‐study offer, the data revealed an almost normal distribution centered around approximately 2 h, with outliers extending to 4 h (Figure 3B). This pattern suggests that while most students preferred shorter, more frequent study sessions, a smaller group opted for longer, intensive periods of study.

The digital self‐study offer, in which students actively self‐enrolled, was utilized by a total of 273 students, representing 39.91% of the 684 students, 345 from the first semester, and 339 from the third semester (Table 1).

**TABLE 1:** Utilization of guided digital and face‐to‐face self‐study offers among first‐ and third‐semester students.

	Guided digital self‐study offer	Guided face‐to‐face self‐study offer	Both	None	Total
First semester, *n* (%)	34 (9.86%)	138 (40.00%)	107 (31.01%)	66 (19.13%)	345 (100%)
Third semester, *n* (%)	84 (24.78%)	56 (16.52%)	48 (14.16%)	151 (44.54%)	339 (100%)
Both semesters, *n* (%)	118 (20.18%)	194 (28.36%)	155 (22.66%)	217 (31.73%)	684 (100%)

**FIGURE 4:**
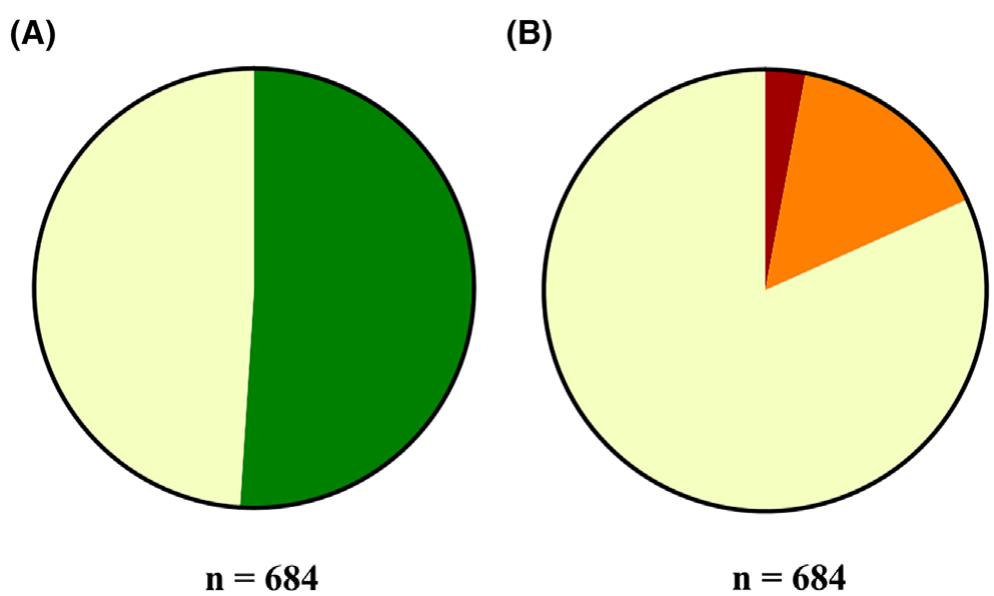
(A) Pie chart illustrating the pattern of usage of the face‐to‐face guided self‐study offer. A total of 51.02% of students (*n* = 684) participated in the face‐to‐face environment (green), while 48.98% did not participate (light yellow). (B) Pie chart showing the pattern of usage in the digital guided self‐study environment. Among the students (*n* = 684), 2.92% participated in the forum (red), 15.35% answered weekly quests (orange), and 81.73% did not participate in the digital environment (light yellow).

The data enables the differentiation of students’ usage behavior, categorizing them into the following groups: students who utilized the guided face‐to‐face self‐study offer (Figure 4A), students who participated in the digital self‐study offer (Figure 4B), students who availed themselves of both options, and students who utilized neither option.

In the first semester, 107 students (31.01%) utilized both the digital self‐study offer and the histology lecture hall. In the third semester, 48 students (14.16%) used both options. Additionally, 66 first‐semester students (19.13%) and 151 third‐semester students (44.54%) utilized neither option. It can furthermore be determined that 559 students (81.73%) from both semesters did not participate in any form in the guided digital self‐study offer. Among those who participated, 20 students (2.92%) engaged in the forum and 105 students (15.35%) completed the weekly quest (Figure 4).

**FIGURE 5:**
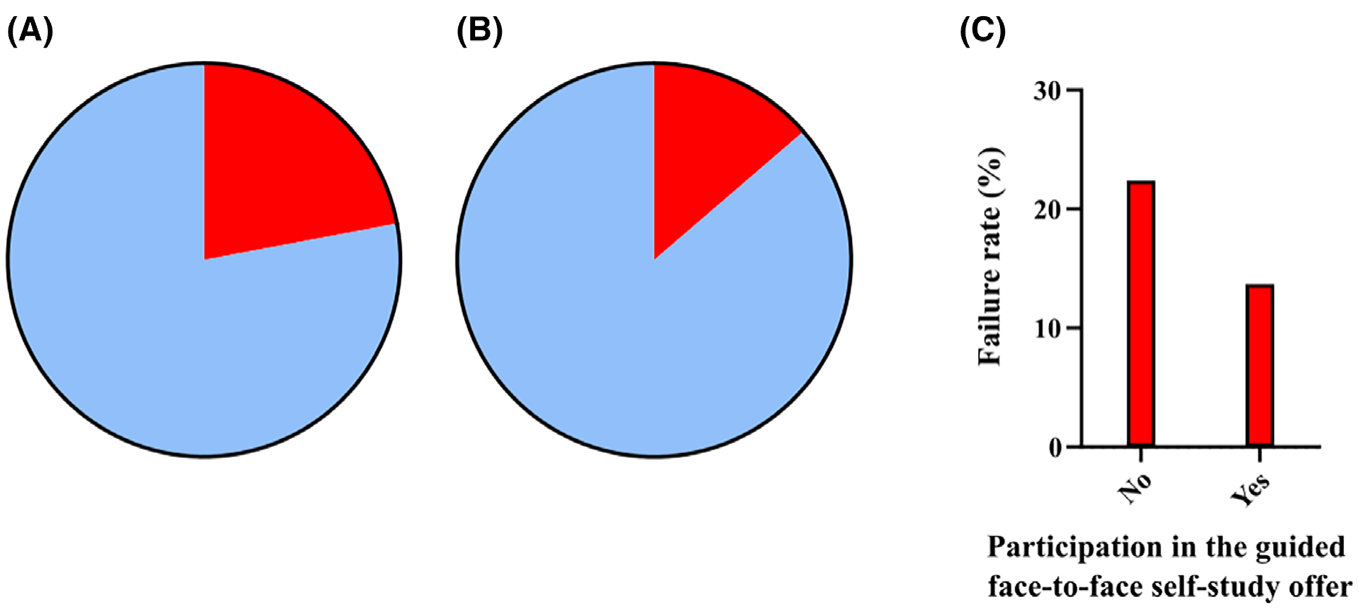
(A) Pie chart depicting the exam success rates of students from both semesters who did not participate in the guided face‐to‐face self‐study offer. Among these students, 81.7% passed the exam (blue), while 18.3% failed (red). (B) Pie chart illustrating the exam success rates of students from both semesters who participated in the guided face‐to‐face self‐study offer. In this group, 94.3% passed the exam (blue), while 5.7% failed (red). (C) Bar chart comparing the failure rates of students from both semesters based on their participation or non‐participation in the guided face‐to‐face self‐study offer. The failure rate was 18.3% for non‐participants and 5.7% for participants.

Identifying a correlation between making use of the face‐to‐face guided self‐study offer and academic performance in the final examination revealed discernible trends. Analysis indicated that students who participated in the guided face‐to‐face self‐study offer had a higher probability of success in the final examination compared to those who did not engage in this supplementary educational offer. The exam questions in histology were analyzed in isolation and evaluated with regard to the pass rate of 60%. For this performance analysis, students were divided into two groups: those who participated in the guided face‐to‐face self‐study offer and those who did not. The comparison of examination results showed that the likelihood of failure was 13.69% for students who participated in the guided face‐to‐face self‐study offer and 22.04% for those who did not engage in the face‐to‐face guided self‐study offer (Figure 5).

The follow‐up questionnaire had a total response rate of 48.83% (334 participants). It was structured into four overarching constructs: Q1—Digital guided self‐study forum (on Moodle), Q2—Digital Microscopy, Q3—Face‐to‐Face guided self‐study offer, and Q4—Histological Sketches. Constructs Q1 to Q3 each consisted of five items, while Q4 consisted of three items. The answer options for all items were: “strongly agree,” “partly agree,” and “disagree” The response frequencies and percentage distributions were analyzed (Table 2).

**TABLE 2:** Distribution of responses across items in the follow‐up questionnaire.

		Disagree, *n* (%)	Partly agree, *n* (%)	Strongly agree, *n* (%)	Total, *n* (%)
*Q1 Digital guided self‐study forum (Moodle)*					
Item 1	I was uncertain about the effectiveness of digital communication in addressing my queries regarding microscopic anatomy	62 (18.563)	209 (62.575)	63 (18.862)	334 (100)
Item 2	I preferred posing questions in person as I anticipated clearer and more direct responses thereby	37 (11.078)	128 (38.323)	169 (50.599)	334 (100)
Item 3	The forum provided ample opportunity for in‐depth discussions on microscopic anatomy	59 (17.665)	221 (66.168)	54 (16.168)	334 (100)
Item 4	Technical barriers or lack of familiarity with the digital forum deterred me from utilizing it	173 (51.796)	112 (33.533)	49 (14.671)	334 (100)
Item 5	I had the impression that my questions were too specific or complex to effectively discuss them in a digital format	142 (42.515)	136 (40.719)	56 (16.766)	334 (100)
*Q2 Digital microscopy*					
Item 1	The digital representation of specimens was of high quality and detailed	9 (2.695)	163 (48.802)	162 (48.503)	334 (100)
Item 2	The software for digital microscopy was intuitive and easy to use	13 (3.892)	119 (35.629)	202 (60.479)	334 (100)
Item 3	I effectively utilized digital specimens to accommodate my own learning pace and needs	6 (1.796)	63 (18.862)	265 (79.341)	334 (100)
Item 4	The ability to annotate and save digital specimens myself enhanced my learning experience	70 (20.958)	147 (44.012)	117 (35.030)	334 (100)
Item 5	Technical issues or limitations hindered my learning experience with digital microscopy	157 (47.006)	144 (43.114)	33 (9.880)	334 (100)
*Q3 Face‐to‐face guided self‐study offer*					
Item 1	The direct interaction with tutors or lecturers in person significantly contributed to my understanding of microscopic anatomy	22 (6.587)	107 (32.036)	205 (61.377)	334 (100)
Item 2	Learning at the microscope in the on‐site histology lecture hall provided me with a more practical and engaging learning experience	16 (4.790)	56 (16.766)	262 (78.443)	334 (100)
Item 3	Engaging in free microscopy sessions in person facilitated exchange and discussion with my peers	13 (3.892)	73 (21.856)	248 (74.251)	334 (100)
Item 4	I found the physical presence in the histology lecture hall more motivating than learning from home	48 (14.371)	130 (38.922)	156 (46.707)	334 (100)
Item 5	The quality and availability of specimens in the on‐site histology lecture hall were superior to the digital alternatives	51 (15.269)	170 (50.898)	113 (33.832)	334 (100)
*Q4 Histological sketches*					
		**No, *n* (%)**	**Yes, *n* (%)**		**Total, *n* (%)**
Item 1	I created my own drawings of histological specimens to deepen my understanding of microscopic anatomy	242 (72.46)	92 (27.54)		334 (100)
	Of those who said yes	**Disagree, *n* (%)**	**Partly agree, *n* (%)**	**Strongly agree, *n* (%)**	**Total, *n* (%)**
Item 2	Creating drawings helped me better recognize and understand important details and structures of the histological specimens	15 (16.30)	55 (59.78)	20 (21.74)	92 (100) Missing 2 (2.17)
Item 3	Generating my own drawings was an effective method for enhancing my long‐term memory of microscopic anatomy	17 (18.48)	51 (55.43)	22 (23.91)	92 (100) Missing 2 (2.17)

**FIGURE 6:**
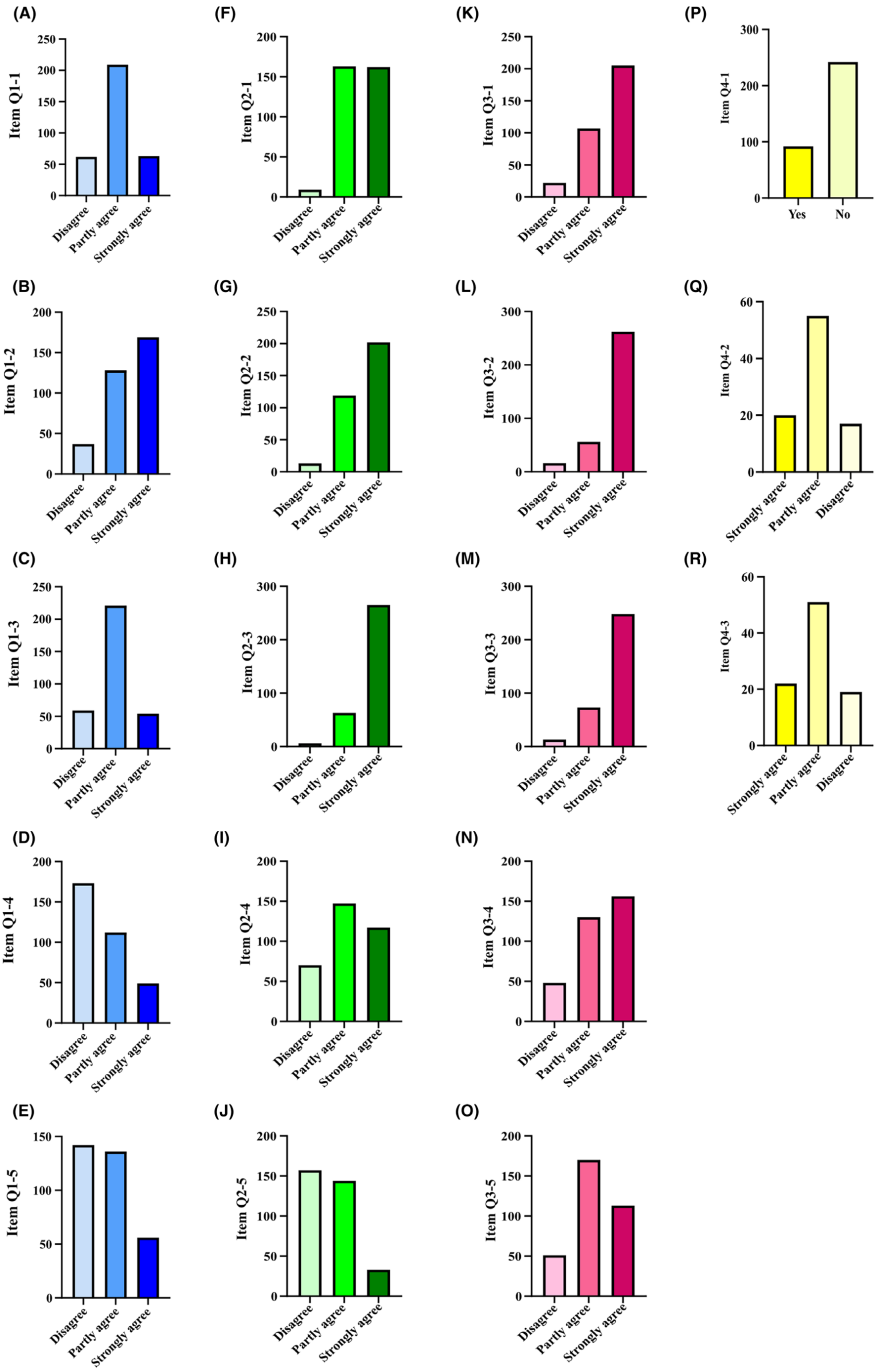
Distribution of responses to follow‐up questionnaire items (Q1–1 to Q4–3), labeled from (A) to (R). Each bar chart represents the response frequencies for individual items, grouped by Likert scale options. Responses are color‐coded as follows: Blue shades for Q1 items (A–E), green shades for Q2 items (F–J), pink shades for Q3 items (K–O), and yellow shades for Q4 items (P–R). The height of each bar indicates the proportion of students selecting a specific response for the corresponding item.

The analysis of the items related to the digital guided self‐study offer (Q1: Digital Forum on Moodle) indicates a general dissatisfaction with this learning environment among students. Notably, approximately 51% (169 students) strongly agreed that they preferred posing questions in person because they anticipated clearer and more direct responses, highlighting a preference for face‐to‐face interaction over digital communication (Figure 6, item Q1–2). Additionally, about 52% (173 students) disagreed with the statement that “Technical barriers or lack of familiarity with the digital forum deterred me from utilizing it,” suggesting that technical difficulties were not the primary issue but rather a lack of effectiveness or preference for digital communication (Figure 6, item Q1–4). Overall, the responses across the Q1 items reflect significant reservations regarding the digitally guided self‐study offer, with many students expressing concerns about the adequacy of digital communication for addressing their learning needs and the perceived limitations of the digital forum in facilitating in‐depth discussions (Figure 6).

The analysis of Q2 Digital Microscopy items suggests that the digital microscopy tools were generally well received by students and provided meaningful support to their learning experience. The responses indicate that the digital microscopy tools were viewed positively, with 60.48% of students (202 out of 334) finding the software intuitive and easy to use, which likely contributed to their engagement with the material (Figure 6, item Q2–2). Additionally, 79.34% of students (265 out of 334) appreciated the ability to learn at their own pace, which allowed for more flexible and individualized study sessions (Figure 6, item Q2–3). The digital representation of specimens was also regarded as high quality and detailed, with 97% of students (325 out of 334) agreeing to some extent with this statement (Figure 6, item Q2–1). Overall, the feedback indicates that the digital microscopy tools were an effective component of the students’ learning resources, particularly in terms of ease of use, flexibility, and the quality of the digital images (Figure 6).

The analysis of Q3, which evaluated the face‐to‐face guided self‐study offer, indicates that this learning environment was strongly favored by students for several key reasons. A significant portion of students, 78.44% (262 out of 334), reported that the hands‐on experience with the microscope in the on‐site histology lecture hall provided a more practical and engaging learning experience, suggesting that the tactile and visual aspects of face‐to‐face learning were particularly valuable (Figure 6, item Q3–2). Additionally, 74.25% of students (248 out of 334) indicated that the opportunity to engage in free microscopy sessions in person facilitated valuable exchanges and discussions with peers, underscoring the importance of social interaction and collaborative learning in this setting (Figure 6, item Q3–3). Furthermore, 61.38% of students (205 out of 334) strongly agreed that direct interaction with tutors or lecturers in person significantly enhanced their understanding of microscopic anatomy, highlighting the value of immediate feedback and personalized instruction (Figure 6, item Q3–1). Moreover, nearly half of the respondents (46.71%, or 156 out of 334) found the physical presence in the histology lecture hall more motivating than studying from home, which points to the positive impact of a dedicated learning environment on student motivation (Figure 6, item Q3–4). Finally, while opinions were more varied on the quality and availability of specimens, 84.73% of students (283 out of 334) acknowledged that the on‐site histology lecture hall provided superior resources compared to digital alternatives, further reinforcing the preference for face‐to‐face learning (Figure 6, item Q3–5).

The evaluation of Q4: Histological Sketches shows that the majority of students (72.46%, or 242 out of 334) chose not to create drawings of histological specimens as part of their study routine (Figure 6, item Q4–1). However, among the 27.54% (92 out of 334) who did engage in drawing, a significant portion found it to be a beneficial practice. Specifically, 81.52% of these students (75 out of 92) reported that creating drawings helped them better recognize and understand important details and structures, with 21.74% (20 out of 92) fully agreeing and 59.78% (55 out of 92) partly agreeing on its usefulness (Figure 6, item Q4–2). Opinions on whether drawing effectively enhanced long‐term memory were somewhat mixed, though a majority still perceived it positively: 55.43% (51 out of 92) partly agreed, and 23.91% (22 out of 92) strongly agreed that it was an effective method, while 18.48% (17 out of 92) disagreed (Figure 6, item Q4–3). Overall, while drawing was not widely adopted by most students, those who did engage in this practice generally found it helpful for deepening their understanding and potentially enhancing memory retention in microscopic anatomy.

## DISCUSSION

The primary aim of this study was to investigate the user behavior of medical students in relation to two distinct learning supplements offered alongside the traditional curriculum in Microscopic Anatomy: a digital guided self‐study offer and a face‐to‐face guided self‐study offer. Our analysis sought to understand which learning environment students preferred and how these preferences impacted their engagement and academic performance. Interestingly, the results revealed a strong preference for the face‐to‐face guided self‐study offer. This preference was clearly demonstrated through usage behavior, with a significantly higher number of students attending the in‐person sessions compared to those who engaged with the digital platform. Specifically, data from the study showed that the average attendance at the face‐to‐face sessions steadily increased as the semester progressed, with 78.44% of students reporting that the practical handling of the microscope in a physical setting was highly beneficial. Furthermore, responses from the follow‐up questionnaire reinforced this preference, with students highlighting the value of direct interaction with peers and tutors, as well as the motivating atmosphere of the histology lecture hall. These findings suggest that, despite the convenience and flexibility of digital learning tools, students heavily favored the tangible, interactive experiences provided by face‐to‐face learning environments. Notably, students who attended the face‐to‐face sessions also performed better in the final examinations, suggesting that the interactive, hands‐on experience provided in these sessions positively influenced their academic success.

During the emergence of the COVID‐19 pandemic, digital learning experienced a significant boost as higher education institutions rapidly shifted to remote education.^22^, ^23^ It was found that the primary resources employed in this transition were videoconferencing tools, educational videos, and virtual platforms, which became essential components of the new digital learning landscape.^24^ In light of recent literature, several compelling findings have emerged that underscore the effectiveness of digital learning environments, particularly during the COVID‐19 pandemic. First, studies have indicated that students’ learning outcomes were significantly higher during e‐learning compared to traditional in‐person education, suggesting that the digital format may offer certain advantages in content delivery and assimilation.^25^ Moreover, this improvement is reflected in the overall students’ average scores, which also saw a significant increase in the e‐learning environment. This trend is further supported by higher engagement ratings in online settings, suggesting that digital platforms may facilitate more effective learning experiences.^26^ Additionally, improved comprehension has been reported with online delivery in certain subjects, likely due to the flexible pace and accessibility of digital resources.^26^ Furthermore, the mean total exam scores were found to be significantly higher among students who participated in blended learning environments—combining both online and in‐person instruction—than among those who only experienced one mode.^27^

Despite these promising findings regarding the effectiveness of digital learning, first concerns began to surface as the initial enthusiasm for online education was tempered by the realities of what quickly came to be known as “Emergency Remote Teaching” (ERT).^28^ The COVID‐19 pandemic abruptly shifted academic institutions from traditional classrooms to ERT. While this change was necessary, it led to the loss of critical hands‐on experiences, increased workloads, and significant curriculum disruptions. Despite a 20% rise in exam scores during ERT, students reported lower engagement, confidence, and perceived learning compared to in‐person classes.^28^ The absence of practical components, such as body donor‐based learning, particularly hindered fields requiring 3D visualization, where the lack of physical interaction diminished understanding.^29^ Although the flexibility in pacing and exams was a positive aspect, it was overshadowed by the challenge of maintaining meaningful student engagement in a predominantly digital environment.^30^ Additionally, the shift to online learning raised concerns about increased anxiety and mental health issues among students, who often felt uncertain about their progress and disconnected from the traditional support systems provided by in‐person education.^30^, ^31^, ^32^ These findings highlight the risks of digital learning when it lacks sufficient interactive, hands‐on components, underscoring the need for a balanced approach in future educational strategies. Psychobiological research further supports these findings by demonstrating that online learning is linked to reduced physiological arousal compared to traditional face‐to‐face learning.^33^, ^34^ Findings have demonstrated that students engaged in online learning exhibit lower cortisol concentrations and higher heart rate variability, indicative of increased parasympathetic activation and a more relaxed physiological state.^34^ In contrast, face‐to‐face learning environments tend to induce higher physiological arousal, which is closely linked to more pronounced emotional expressions, such as enjoyment.^34^ This heightened emotional engagement has been correlated with improved academic performance,^35^ suggesting that the dynamic and interactive nature of in‐person learning not only fosters deeper learning but also contributes to students’ overall academic success.

Building on the synthesis of both perspectives on digital learning, we introduce the concept of the ‘Contextual Digital Divide’ to describe a gap not in access to technology,^21^ but in the effectiveness of digital education in meeting specific educational needs. Traditionally, the digital divide refers to the disparity between those with and without access to technology, leading to unequal learning opportunities.^21^, ^36^ However, our research reveals another dimension: the situations where digital learning falls short compared to face‐to‐face interactions. Our findings substantiate this concept by showing that when students have a choice, they overwhelmingly prefer face‐to‐face learning in some situations, as evidenced by our quantitative analysis of usage behavior. This preference highlights the limitations of digital platforms in replicating the depth of engagement and hands‐on experiences that traditional in‐person learning provides, particularly in some educational cases. This underscores the need to address this contextual gap in educational strategies, while recognizing that face‐to‐face teaching may not always be the best option in every scenario.

A significant majority of students concurred with the statement, “I preferred posing questions in person as I anticipated clearer and more direct responses thereby,” a preference that is corroborated by existing research, which underscores the critical role of in‐person communication.^37^ Notably, a substantial majority of students agreed that “Learning at the microscope in the on‐site histology lecture hall provided me with a more practical and engaging learning experience,” a sentiment that aligns with research demonstrating that hands‐on, in‐person learning environments not only enhance practical skills and engagement but also have a positive effect on theoretical knowledge—an aspect that remains vital in the digital age.^38^ It could be argued that the process of physically handling the microscope, adjusting focus, and directly observing specimens helps students develop technical proficiency and an intuitive understanding of spatial and structural relationships that may be difficult to achieve with digital tools alone. Similar to research demonstrating that longhand note‐taking enhances deeper learning compared to typing on laptops,^39^ the hands‐on use of microscopes encourages active engagement and deeper cognitive processing, as opposed to the more passive interaction often associated with digital methods. Furthermore, this immersive learning environment promotes active involvement and provides immediate opportunities for interaction with peers and tutors, amplifying its educational impact—a dynamic that is clearly reflected in our empirical data, where students rated these aspects of the learning experience overwhelmingly positively. In contrast to digital specimens, where annotations are typically pre‐defined, the hands‐on course at the microscope encourages students to develop their own annotations or at least generate ideas for them. This difference warrants careful didactic consideration, as the optimal learning strategy regarding this aspect remains subject to debate. Interestingly, a substantial majority of students also agreed that “Engaging in free microscopy sessions in person facilitated exchange and discussion with my peers,” a finding that aligns well with existing research on Peer Instruction.^40^, ^41^, ^42^ This approach, which encourages students to discuss and clarify concepts among themselves, is recognized for its ability to deepen comprehension and foster a more interactive learning environment. Additionally, Peer Instruction has been shown to enhance meaningful learning, particularly in improving students’ ability to solve novel problems, further highlighting its effectiveness as a valuable educational tool.^43^ In addition to the value of peer instruction, which promotes active discussion among students, direct communication with near‐peer tutors provides unique benefits. Near‐peer tutoring creates a safe and supportive learning environment, enabling students to engage more confidently while receiving tailored coaching and socio‐emotional support.^44^ Tutors themselves benefit from improved teaching confidence and learning regulation, as well as enhanced feedback and communication skills.^45^ The dual pedagogical value of near‐peer teaching emerges as a standout feature in modern educational environments, as it not only encourages academic and personal growth for both learners and tutors but also complements traditional expert‐guided instruction. Peer and near‐peer discussions not only facilitate collaborative learning but also serve as a mechanism for generating self‐assessment and feedback, both of which are key drivers of deep learning.^46^ By actively engaging in reflective dialogue, students enhance their understanding and develop critical thinking skills, ultimately fostering deeper, more sustainable learning outcomes.^47^

The digital microscope received highly favorable evaluations from the students, with its benefits accessible to all participants, regardless of whether they engaged in the guided face‐to‐face learning or the digital offering. Among the highlighted advantages, students particularly appreciated the ability to effectively utilize digital specimens to accommodate their own learning pace and needs—a convenience often cited as a key strength of digital learning.^48^ Despite the widespread appreciation for this digital tool, our analysis revealed a considerable increase in performance among students who participated in the face‐to‐face guided self‐study offer. This finding aligns with existing literature, which suggests that combining face‐to‐face instruction with online resources can enhance retention and deepen understanding.^49^ Therefore, while our research does not discourage the use of digital learning, it strongly supports the reinforcement of face‐to‐face teaching, which can be supplemented by digital learning applications. This study provides insights for addressing the Contextual Digital Divide in modern tertiary anatomy education. While digital tools offer valuable flexibility and accessibility, particularly for foundational learning and revision, they should not replace the essential face‐to‐face components of anatomy education. The in‐person setting uniquely addresses learning needs that digital formats cannot fully replicate, such as hands‐on engagement with microscopes, immediate feedback from tutors, and collaborative peer interactions. These elements are indispensable for fostering technical proficiency, critical thinking, and deeper conceptual understanding. To maintain a balanced approach, institutions should ensure that digital resources support and enhance, rather than overshadow, the irreplaceable benefits of face‐to‐face learning in anatomy education.

Taking this entire project into account, several avenues for future research emerge that could further deepen our understanding of the Contextual Digital Divide. A critical area of exploration is identifying additional scenarios within health sciences education where the Contextual Digital Divide becomes apparent. Understanding these contexts can guide the development of strategies to adequately address and overcome the limitations of digital learning in these specific settings. Moreover, future studies could investigate the specific conditions under which digital tools can most effectively supplement traditional teaching methods, focusing on how to optimize the balance between digital flexibility and the benefits of direct, in‐person interaction.

While our return rate of approximately 50% aligns with findings from a comprehensive generalizability analysis of online student evaluations—recommending a minimum response rate of 48% to ensure acceptable precision^50^—future studies should aim to achieve even higher participation rates. Larger response rates would not only enhance the representativeness of findings but also provide a broader foundation for exploring diverse student perspectives and behaviors in depth. Additionally, research could be extended to explore the psychobiological effects of different learning modalities on student well‐being and stress levels, with an emphasis on how these factors influence learning outcomes and competencies in the long run. Finally, a broader cross‐disciplinary analysis could help identify whether the Contextual Digital Divide observed in this study is prevalent in other fields of study, and how educational strategies can be adapted to mitigate it across various academic disciplines.

## CONCLUSION

In conclusion, this study highlights the key aspects of what we term the “Contextual Digital Divide,” wherein digital learning may not fully meet the needs of students in certain educational contexts. In our research, students were given the freedom to choose between a digital guided self‐study offer and a face‐to‐face guided self‐study offer. The majority of students opted for the face‐to‐face option, a choice that was associated with improved academic performance. The key factors influencing this preference included the value of direct interactions with tutors, the more practical and engaging learning experiences provided by in‐person sessions, the opportunity for immediate exchange and discussion with peers, and a higher level of motivation compared to studying alone at home. These findings underscore the importance of preserving and enhancing face‐to‐face educational opportunities, particularly in scenarios where direct, hands‐on learning is critical to student success. These findings highlight the need to maintain and strengthen face‐to‐face learning opportunities, especially in situations where direct, hands‐on experiences are essential.

## AUTHOR CONTRIBUTIONS

**Morris Gellisch:** Conceptualization; methodology; software; data curation; investigation; validation; formal analysis; supervision; visualization; project administration; writing – original draft; writing – review and editing. **Juliane Cramer:** Software; data curation; validation; formal analysis; visualization; writing – review and editing. **Justin Trenkel:** Data curation; investigation; validation; formal analysis; visualization. **Franziska Bäker:** Writing – review and editing; formal analysis; validation. **Martin Bablok:** Writing – review and editing; formal analysis; data curation. **Gabriela Morosan‐Puopolo:** Validation; writing – review and editing; conceptualization. **Thorsten Schäfer:** Conceptualization; data curation; validation; resources; writing – review and editing; formal analysis. **Beate Brand‐Saberi:** Conceptualization; data curation; validation; formal analysis; resources; writing – review and editing.

## CONFLICT OF INTEREST STATEMENT

The authors have no conflicts of interest to declare.

## DECLARATION OF GENERATIVE AI AND AI‐ASSISTED TECHNOLOGIES IN THE WRITING PROCESS

During the preparation of this work, the authors used ChatGPT 4.0 in order to enhance the readability and language of this work. After using this tool, the authors reviewed and edited the content as needed and took full responsibility for the content of the publication.

## Supporting information


**Appendix S1.** Interface of the digital guided self‐study offer available on the Moodle learning management platform. The interface provides options for uploading materials, such as annotated tissue section images, and includes a feature for students to directly ask questions to tutors.

**Appendix S2.** Extended view of the interface of the digital guided self‐study offer on the Moodle learning management platform. This view includes an additional digital tool, the Histo‐Challenge of the Week, where students can submit their answers to the weekly histology question, developed by the tutors.

Text S1.

## APPENDIX A

Representative histological drawings created by students, illustrating varying levels of detail and annotation. Each drawing includes labeled structures and reflects individual differences in the depth of histological depiction and accuracy.

## APPENDIX B

Examples of typical questions from the final exams in Microscopic Anatomy (Semester 1 and Semester 3). The figure presents a selection of exam questions in their original German version alongside their English translation, illustrating the scope and level of assessment in the respective semesters.

**Table:**

Semester	Example of a typical exam question	English translation
1	**Um welches Gewebe handelt es sich bei der Abbildung? Wählen Sie die richtige Antwort** Plurivakuoläres Fettgewebe Elastisches Band Myokard Muköse Drüse Sehne im Querschnitt	**Which type of tissue is shown in the image? Select the correct answer** Multivacuolar adipose tissue Elastic band Myocardium Mucous gland Tendon in cross‐section
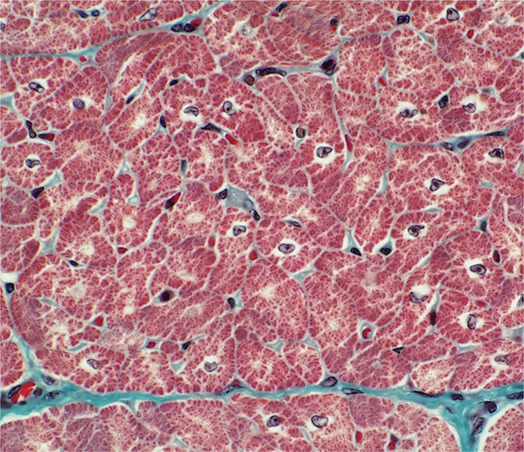
1	**Welche Aussage zur Abbildung ist richtig?** Das abgebildete Gewebe enthält Kollagen Typ I Das abgebildete Gewebe wird von alpha‐Motoneuronen innerviert Das abgebildete Gewebe besitzt Disci intercalares Das abgebildete Gewebe besitzt Z‐Scheiben Das abgebildete Gewebe kann im kontrahierten Zustand verharren	**Which statement about the image is correct?** The depicted tissue contains Type I collagen The depicted tissue is innervated by alpha motor neurons The depicted tissue has intercalated discs (Disci intercalares) The depicted tissue has Z‐discs (Z‐Scheiben) The depicted tissue can remain in a contracted state
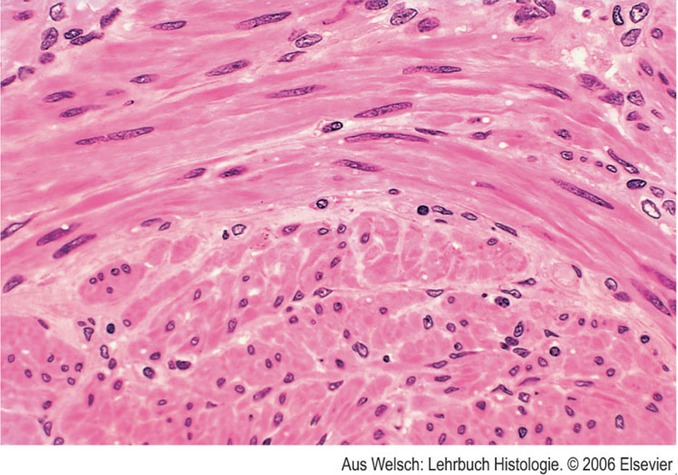
1	**Woran ist unter anderem eine muköse Drüsenzelle zu erkennen?** Am runden, zentral gelegenen Zellkern Am platten, basal gelegenen Zellkern An einer apikalen Granulierung An basal gelegenen Myoepithelzellen Am im Lumen enthaltenen Schleimpfropf	**How can a mucous gland cell be identified, among other characteristics?** By its round, centrally located nucleus By its flat, basally located nucleus By its apical granulation By its basally located myoepithelial cells By the mucous plug contained in the lumen
3	**Die Funktion der Blut‐Harnschranke ist lebenswichtig. Sie besteht aus den folgenden Strukturen:** Mesangiumzellen, Kapillarendothel und Bowman‐Kapsel Kapillarendothel, juxtaglomeruläre Zellen, Basalmembran Macula densa, juxtaglomeruläre Zellen, Podozyten Kapillarendothel mit Diaphragma und Basalmembran **E**. Kapillarendothel, Basalmembran, Podozytenfüße	**The function of the blood‐urine barrier is essential for life. It consists of the following structures:** Mesangial cells, capillary endothelium, and Bowman's capsule Capillary endothelium, juxtaglomerular cells, and basement membrane Macula densa, juxtaglomerular cells, and podocytes Capillary endothelium with diaphragm and basement membrane Capillary endothelium, basement membrane, and podocyte feet
3	**Welche Aussage zum juxtaglomerulären Apparat der Niere ist richtig?** Er dient der Filtration des Primärharns Er besteht aus Podozyten und Mesangiumzellen Er dient der tubuloglomerulären Rückkopplung Er dient der Regeneration von Endothelzellen Er umfasst Teile des proximalen Tubulus	**Which statement about the juxtaglomerular apparatus of the kidney is correct?** It is involved in the filtration of primary urine It consists of podocytes and mesangial cells It is responsible for tubuloglomerular feedback It serves the regeneration of endothelial cells It includes parts of the proximal tubule
3	**Welche Aussage zu glatten Muskelzellen ist richtig?** Aktin und Myosin sind an Dense Bodies verankert Glatte Muskelzellen verfügen über motorische Endplatten Glatte Muskelzellen besitzen keine Basalmembranen Beim Multi‐Unit‐Typ sind die Zellen untereinander durch Gap Junctions gekoppelt Glatte Muskelzellen kontrollieren in den Kapillaren den peripheren Gefäßwiderstand	**Which statement about smooth muscle cells is correct?** Actin and myosin are anchored to dense bodies Smooth muscle cells have motor endplates Smooth muscle cells lack basement membranes In the multi‐unit type, the cells are connected via gap junctions Smooth muscle cells control peripheral vascular resistance in capillaries

## References

[1] Carneiro BD , Pozza DH , Tavares I . Perceptions of medical students towards the role of histology and embryology during curricular review. BMC Med Educ. 2023;23:74. 10.1186/s12909-023-04019-4 36717846 PMC9885397

[2] Das M , Ettarh R , Lowrie DJ , Rengasamy P , Lee LMJ , Williams JM , et al. A guide to competencies, educational goals, and learning objectives for teaching medical histology in an undergraduate medical education setting. Med Sci Educ. 2019;29:523–534. 10.1007/s40670-018-00688-9 34457510 PMC8368454

[3] Murchan P , Ó'Brien C , O'Connell S , McNevin CS , Baird A‐M , Sheils O , et al. Deep learning of histopathological features for the prediction of tumour molecular genetics. Diagnostics. 2021;11:1406. 10.3390/diagnostics11081406 34441338 PMC8393642

[4] Wu Y , Cheng M , Huang S , Pei Z , Zuo Y , Liu J , et al. Recent advances of deep learning for computational histopathology: principles and applications. Cancers (Basel). 2022;14:1199. 10.3390/cancers14051199 35267505 PMC8909166

[5] Cullen MW , Geske JB , Anavekar NS , McAdams JA , Beliveau ME , Ommen SR , et al. Reinvigorating continuing medical education: meeting the challenges of the digital age. Mayo Clin Proc. 2019;94:2501–2509. 10.1016/j.mayocp.2019.07.004 31806103

[6] Kuhn S , Frankenhauser S , Tolks D . Digitale Lehr‐ und Lernangebote in der medizinischen Ausbildung. Bundesgesundheitsblatt Gesundheitsforschung Gesundheitsschutz. 2018;61:201–209. 10.1007/s00103-017-2673-z 29234823

[7] Egarter S , Mutschler A , Brass K . Impact of COVID‐19 on digital medical education: compatibility of digital teaching and examinations with integrity and ethical principles. Int J Educ Integr. 2021;17:18. 10.1007/s40979-021-00084-8

[8] Gaur U , Majumder MAA , Sa B , Sarkar S , Williams A , Singh K . Challenges and opportunities of preclinical medical education: COVID‐19 crisis and beyond. SN Compr Clin Med. 2020;2:1992–1997. 10.1007/s42399-020-00528-1 32984766 PMC7508422

[9] Hayat AA , Keshavarzi MH , Zare S , Bazrafcan L , Rezaee R , Faghihi SA , et al. Challenges and opportunities from the COVID‐19 pandemic in medical education: a qualitative study. BMC Med Educ. 2021;21:247. 10.1186/s12909-021-02682-z 33926439 PMC8082480

[10] Chapman JA , Lee LMJ , Swailes NT . From scope to screen: the evolution of histology education. Adv Exp Med Biol. 2020;1260:75–107. 10.1007/978-3-030-47483-6_5 33211308

[11] Tauber Z , Lichnovska R , Erdosova B , Zizka R , Cizkova K . Modernized didactic techniques and their impact on the teaching of histology by means of virtual microscopy. Interdiscip J Virtual Learn Med Sci. 2021;12:162–168.

[12] Xu C . Is virtual microscopy really better for histology teaching? Anat Sci Educ. 2013;6:138. 10.1002/ase.1337 23184604

[13] Joaquim DC , Hortsch M , da Silva ASR , David PB , de Leite ACRM , Girão‐Carmona VCC . Digital information and communication technologies on histology learning: what to expect?—an integrative review. Anat Histol Embryol. 2022;51:180–188. 10.1111/ahe.12776 34921436

[14] Alsaid B , Bertrand M . Students' memorization of anatomy, influence of drawing. Morphologie. 2016;100:2–6. 10.1016/j.morpho.2015.11.001 26775609

[15] Khapre M , Deol R , Sharma A , Badyal D . Near‐peer tutor: a solution for quality medical education in faculty constraint setting. Cureus. 2021;13(7):e16416. 10.7759/cureus.16416 34422460 PMC8369978

[16] Shenoy A , Petersen KH . Peer tutoring in preclinical medical education: a review of the literature. Med Sci Educ. 2020;30:537–544. 10.1007/s40670-019-00895-y 34457698 PMC8368558

[17] Gellisch M , Morosan‐Puopolo G , Wolf OT , Moser DA , Zaehres H , Brand‐Saberi B . Interactive teaching enhances students' physiological arousal during online learning. Ann Anat. 2023;247:152050. 10.1016/j.aanat.2023.152050 36693546

[18] Cogdell B , Torsney B , Stewart K , Smith RA . Technological and traditional drawing approaches encourage active engagement in histology classes for science undergraduates. Biosci Educ. 2012;19:1–15. 10.11120/beej.2012.19000003

[19] Balemans MCM , Kooloos JGM , Donders ART , Van der Zee CEEM . Actual drawing of histological images improves knowledge retention. Anat Sci Educ. 2016;9:60–70. 10.1002/ase.1545 26033842

[20] Rafi A , Anwar MI , Manzoor S , Anwar S . Drawing is an important tool to learn context‐based histology in an integrated undergraduate medical curriculum. J Taibah Univ Med Sci. 2023;18:886–893. 10.1016/j.jtumed.2023.01.005 36852236 PMC9957768

[21] Wei K‐K , Teo H‐H , Chan HC , Tan BCY . Conceptualizing and testing a social cognitive model of the digital divide. Inf Syst Res. 2011;22:170–187. 10.1287/isre.1090.0273

[22] Aristovnik A , Karampelas K , Umek L , Ravšelj D . Impact of the COVID‐19 pandemic on online learning in higher education: a bibliometric analysis. Front Educ. 2023;8:1225834. 10.3389/feduc.2023.1225834

[23] Ebner M , Schön S , Braun C , Ebner M , Grigoriadis Y , Haas M , et al. COVID‐19 Epidemic as E‐Learning Boost? Chronological Development and Effects at an Austrian University against the Background of the Concept of “E‐Learning Readiness.” Future Internet. 2020;12:94. 10.3390/fi12060094

[24] Rodríguez ML , Pulido‐Montes C . Use of digital resources in higher education during COVID‐19: a literature review. Educ Sci. 2022;12:612. 10.3390/educsci12090612

[25] Mastour H , Emadzadeh A , Hamidi Haji Abadi O , Niroumand S . Are students performing the same in E‐learning and in‐person education? An introspective look at learning environments from an Iranian medical school standpoint. BMC Med Educ. 2023;23:209. 10.1186/s12909-023-04159-7 37016360 PMC10072012

[26] Kieran NW , Sinnott JF , Shah YB , Patil S , Round KJ , Wenyon S , et al. Comparison of online to in‐person administration of a medical student leadership curriculum. Acad Med. 2023;98:S206–S207. 10.1097/ACM.0000000000005408

[27] Bazrgar A , Rahmanian M , Ghaedi A , Heidari A , Bazrafshan M , Amini M , et al. Face‐to‐face, online, or blended: which method is more effective in teaching electrocardiogram to medical students. BMC Med Educ. 2023;23:566. 10.1186/s12909-023-04546-0 37559020 PMC10413712

[28] Wilhelm J , Mattingly S , Gonzalez VH . Perceptions, satisfactions, and performance of undergraduate students during Covid‐19 emergency remote teaching. Anat Sci Educ. 2022;15:42–56. 10.1002/ASE.2161 34859608 PMC9011711

[29] Asante EA , Maalman RS , Ali MA , Donkor YO , Korpisah JK . Perception and attitude of medical students towards cadaveric dissection in anatomical science education. Ethiop J Health Sci. 2021;31:867–874. 10.4314/ejhs.v31i4.22 34703187 PMC8512940

[30] Cuschieri S , Calleja Agius J . Spotlight on the shift to remote anatomical teaching during Covid‐19 pandemic: perspectives and experiences from the University of Malta. Anat Sci Educ. 2020;13:671–679. 10.1002/ase.2020 32956579 PMC7537517

[31] Chang W , Shi L , Zhang L , Jin Y , Yu J . The mental health status and associated factors among medical students engaged in online learning at home during the pandemic: a cross‐sectional study from China. Front Psychiatry. 2021;12:755503. 10.3389/fpsyt.2021.755503 35002796 PMC8732944

[32] Gellisch M , Bablok M , Morosan‐Puopolo G , Schäfer T , Brand‐Saberi B . Dynamically changing mental stress parameters of first‐year medical students over the three‐year course of the COVID‐19 pandemic: a repeated cross‐sectional study. Healthcare. 2023;11:1558. 10.3390/healthcare11111558 37297698 PMC10252315

[33] Gellisch M , Bablok M , Brand‐Saberi B , Schäfer T . Neurobiological stress markers in educational research: a systematic review of physiological insights in health science education. Trends Neurosci Educ. 2024;37:100242. 10.1016/j.tine.2024.100242 39638492

[34] Gellisch M , Wolf OT , Minkley N , Kirchner WH , Brüne M , Brand‐Saberi B . Decreased sympathetic cardiovascular influences and hormone‐physiological changes in response to Covid‐19‐related adaptations under different learning environments. Anat Sci Educ. 2022;15:811–826. 10.1002/ase.2213 35968688

[35] Puca RM , Schmalt H‐D . Task enjoyment: a mediator between achievement motives and performance. Motiv Emot. 1999;23:15–29. 10.1023/A:1021327300925

[36] van de Werfhorst HG , Kessenich E , Geven S . The digital divide in online education: inequality in digital readiness of students and schools. Comput Educ Open. 2022;3:100100. 10.1016/j.caeo.2022.100100 PMC9435462 40478103

[37] Stieger S , Lewetz D , Willinger D . Face‐to‐face more important than digital communication for mental health during the pandemic. Sci Rep. 2023;13:8022. 10.1038/s41598-023-34957-4 37198196 PMC10191089

[38] Van Ryneveld L , Holm DE , Cronje T , Leask R . The impact of practical experience on theoretical knowledge at different cognitive levels. J S Afr Vet Assoc. 2020;91:1–7. 10.4102/jsava.v91i0.2042 PMC7433234 32787421

[39] Mueller PA , Oppenheimer DM . The pen is mightier than the keyboard. Psychol Sci. 2014;25:1159–1168. 10.1177/0956797614524581 24760141

[40] Damon W . Peer education: the untapped potential. J Appl Dev Psychol. 1984;5:331–343. 10.1016/0193-3973(84)90006-6

[41] Lucas A . Using peer instruction and i‐clickers to enhance student participation in calculus. PRIMUS. 2009;19:219–231. 10.1080/10511970701643970

[42] Tullis JG , Goldstone RL . Why does peer instruction benefit student learning? Cogn Res Princ Implic. 2020;5:15. 10.1186/s41235-020-00218-5 32274609 PMC7145884

[43] Cortright RN , Collins HL , DiCarlo SE . Peer instruction enhanced meaningful learning: ability to solve novel problems. Adv Physiol Educ. 2005;29:107–111. 10.1152/advan.00060.2004 15905155

[44] Alexander SM , Dallaghan GLB , Birch M , Smith KL , Howard N , Shenvi CL . What makes a near‐peer learning and tutoring program effective in undergraduate medical education: a qualitative analysis. Med Sci Educ. 2022;32:1495–1502. 10.1007/s40670-022-01680-0 36415502 PMC9672576

[45] Zhu MM , Brennan MT . Near‐peer perspectives on a voluntary peer teaching program: challenges and solutions. Med Sci Educ. 2024;34:1–28. 10.1007/s40670-024-02148-z PMC11933544 40144123

[46] Lynch R , McNamara PM , Seery N . Promoting deep learning in a teacher education programme through self‐ and peer‐assessment and feedback. Eur J Teach Educ. 2012;35:179–197. 10.1080/02619768.2011.643396

[47] Kovač VB , Nome DØ , Jensen AR , Skreland LL . The why, what and how of deep learning: critical analysis and additional concerns. Educ Inq. 2023;14:1–17. 10.1080/20004508.2023.2194502

[48] Müller C , Mildenberger T . Facilitating flexible learning by replacing classroom time with an online learning environment: a systematic review of blended learning in higher education. Educ Res Rev. 2021;34:100394. 10.1016/j.edurev.2021.100394

[49] Terry VR , Terry PC , Moloney C , Bowtell L . Face‐to‐face instruction combined with online resources improves retention of clinical skills among undergraduate nursing students. Nurse Educ Today. 2018;61:15–19. 10.1016/j.nedt.2017.10.014 29153453

[50] Gerbase MW , Germond M , Cerutti B , Vu NV , Baroffio A . How many responses do we need? Using generalizability analysis to estimate minimum necessary response rates for online student evaluations. Teach Learn Med. 2015;27:395–403. 10.1080/10401334.2015.1077126 26507997

