# Supplementary figures and images for: Introducing the contextual digital divide: Insights from microscopic anatomy on usage behavior and effectiveness of digital versus face‐to‐face learning
Source: Anat Sci Educ. 2025 Feb 28;18(4):347–64. doi: 10.1002/ase.70010 (PMC11960422; doi:10.1002/ase.70010)

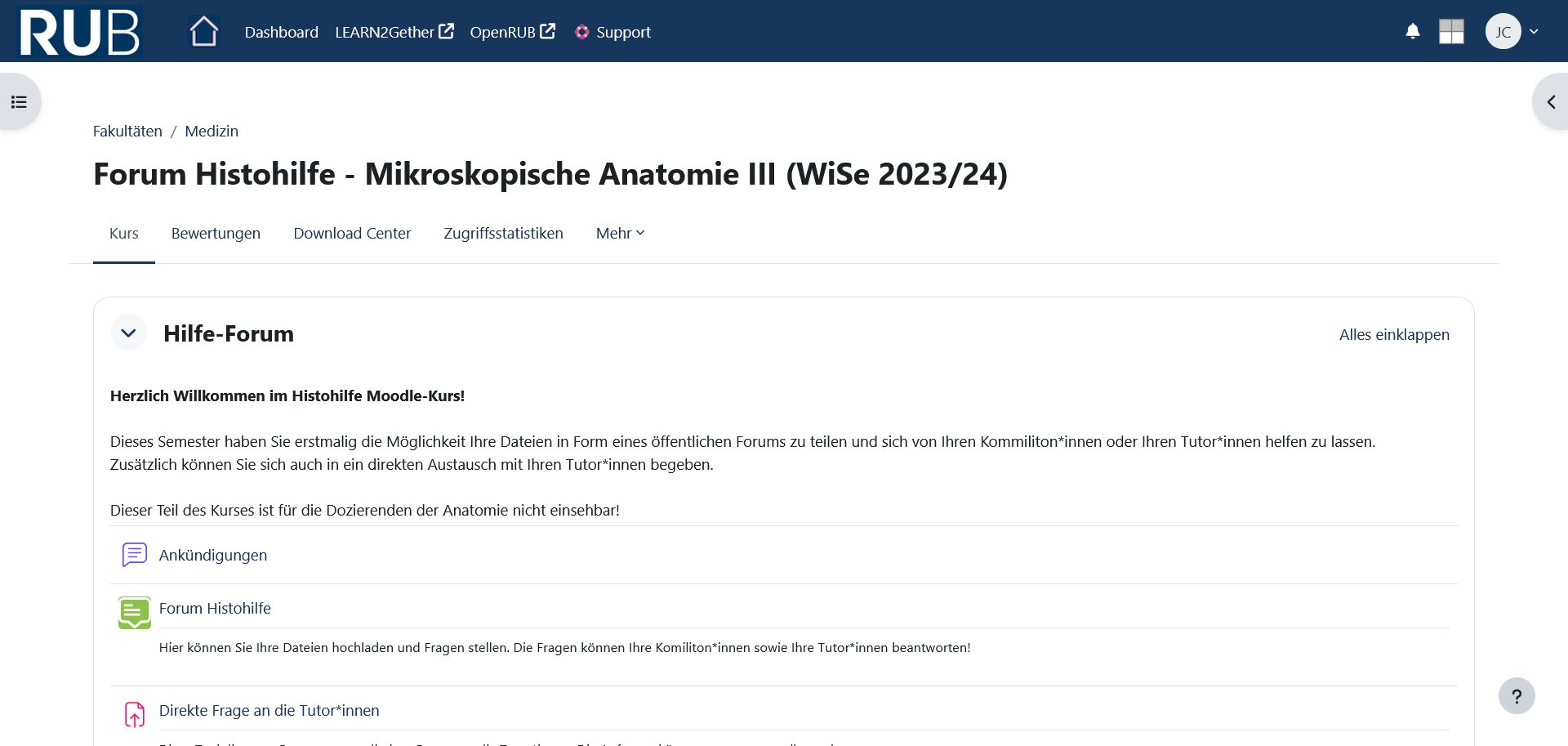

Supplement: Supplementary file 1 — Appendix S1. Interface of the digital guided self‐study offer available on the Moodle learning management platform. The interface provides options for uploading materials, such as annotated tissue section images, and includes a feature for students to directly ask questions to tutors. [file ASE-18-347-s001.png]

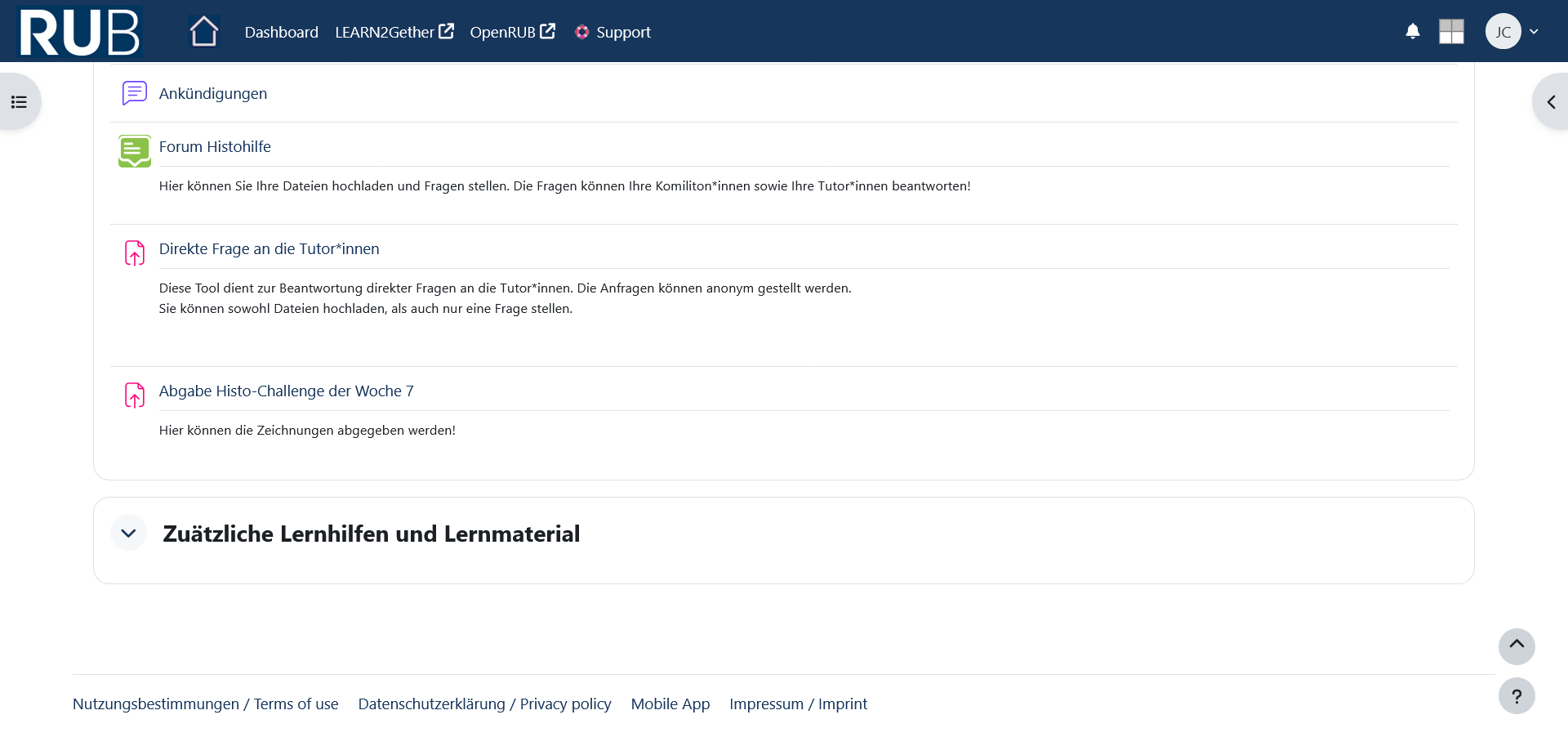

Supplement: Supplementary file 2 — Appendix S2. Extended view of the interface of the digital guided self‐study offer on the Moodle learning management platform. This view includes an additional digital tool, the Histo‐Challenge of the Week, where students can submit their answers to the weekly histology question, developed by the tutors. [file ASE-18-347-s003.png]
