# Supplementary material for: Introducing the contextual digital divide: Insights from microscopic anatomy on usage behavior and effectiveness of digital versus face‐to‐face learning
Source: Anat Sci Educ. 2025 Feb 28;18(4):347–64. doi: 10.1002/ase.70010 (PMC11960422; doi:10.1002/ase.70010)
Supplement: Supplementary file 3 — Text S1. [file ASE-18-347-s002.docx]

Appendix S1: Interface of the digital guided self-study offer available on the Moodle learning management platform. The interface provides options for uploading materials, such as annotated tissue section images, and includes a feature for students to directly ask questions to tutors.

Appendix S2: Extended view of the interface of the digital guided self-study offer on the Moodle learning management platform. This view includes an additional digital tool, the Histo-Challenge of the Week, where students can submit their answers to the weekly histology question, developed by the tutors.
